# Trip‐Based Measures of Naturalistic Driving: Exploring connections to older adult drivers with respect to amyloid‐positive and amyloid‐negative

**DOI:** 10.1002/alz70857_103469

**Published:** 2025-12-25

**Authors:** Alper Kursat Uysal, Sai Shanmukh Varma Rudraraju, Sai Santosh Reddy Danda, Yi Lu Murphy, Amanda Cook Maher, Carol C Persad, Savannah G Rose, Robert A. Koeppe, Bruno Giordani

**Affiliations:** ^1^ University of Michigan‐Dearborn, Dearborn, MI, USA; ^2^ Alanya Alaaddin Keykubat University, Alanya, Antalya, Turkey; ^3^ University of Michigan ‐ Dearborn, Dearborn, MI, USA; ^4^ University of Michigan, Ann Arbor, MI, USA; ^5^ University of Michigan Medical School, Ann Arbor, MI, USA

## Abstract

**Background:**

Identifying subtle changes in cognitively demanding tasks for older persons, such as daily driving, may reveal connections to early signs of cognitive decline associated with Alzheimer's disease (AD). Research has shown that brain amyloid positivity is strongly associated with AD. This study analyzes various trip‐based quantitative measures derived from naturalistic driving to distinguish older adults with amyloid‐positivity (Aβ+) from those who are amyloid‐negative (Aβ‐). Significant trip‐based naturalist driving attributes identified in this study will be used in future development of machine learning models for classifying Aβ+ and Aβ‐ participants.

**Method:**

We analyzed naturalistic driving data from 30 Aβ+ (2,577 trips) and 30 Aβ‐ (2,533 trips) consensus diagnosed participants. An internet facilitated data acquisition system was installed in each participant's vehicle, and driving trips were recorded over approximately one month between 2021 and 2024. The mean ages of the Aβ+ and Aβ‐ groups were 72.9±4.2 and 71.4±5.6 respectively. Each recorded trip included three types of data—videos, vehicle signals, and physiological signals. We studied 22 trip‐based attributes, some of which had not been examined previously in the research community, including attributes derived from valid and invalid trips. A valid trip contains all required data signals, while an invalid trip has one or more signals missing. Statistical *t*‐tests (α=0.05) were used to evaluate the quantitative attributes of the two groups.

**Result:**

The following attributes were found to be significant: Aβ‐ drivers had a higher percentage of valid trips (*p* = 0.026), while Aβ+ drivers had longer average trip duration (*p* = 0.037), longer average trip distance (*p* = 0.049), and a higher percentage of weekday night trips (*p* = 0.048).

**Conclusion:**

Our research demonstrated that Aβ‐ participants had higher percentage of valid trips, potentially indicating better capabilities to operate the data acquisition devices. The longer average trip durations and distances observed in Aβ+ participants may reflect less efficient route choices while driving. Additionally, the higher percentage of weekday night trips among Aβ+ participants suggests higher potential risk of more nighttime driving activity.

